# Efficient Fault Detection of Rotor Minor Inter-Turn Short Circuit in Induction Machines Using Wavelet Transform and Empirical Mode Decomposition

**DOI:** 10.3390/s23167109

**Published:** 2023-08-11

**Authors:** Attiq Ur Rehman, Weidong Jiao, Jianfeng Sun, Muhammad Sohaib, Yonghua Jiang, Mahnoor Shahzadi, Muhammad Ijaz Khan

**Affiliations:** 1 School of Computer Science and Technology, Zhejiang Normal University, Jinhua 321004, China; atnutkani@zjnu.edu.cn (A.U.R.); sohaibdurrani@zjnu.edu.cn (M.S.); 2 Zhejiang Institute of Photoelectronics & Zhejiang Institute for Advanced Light Source, Zhejiang Normal University, Jinhua 321004, China; 3 Key Laboratory of Intelligent Operation and Maintenance Technology & Equipment for Urban Rail Transit of Zhejiang Province, Jinhua 321004, China; sunjf1991@zjnu.edu.cn; 4 School of Engineering, Zhejiang Normal University, Jinhua 321004, China; 5 Xingzhi College, Zhejiang Normal University, Lanxi 321100, China; 6 School of Information and Communication Engineering, University of Electronic Science and Technology, Chengdu 610054, China; mahnoor.gr8@gmail.com; 7 Institute of Mechanical & Manufacturing Engineering, Khwaja Fareed University of Engineering & Information Technology, Rahim Yar Khan 64200, Pakistan; ijaz.khan@kfueit.edu.pk

**Keywords:** doubly fed induction generator, empirical mode decomposition, inter-turn short-circuit fault, rotor winding, wavelet transformation

## Abstract

This paper introduces a novel approach for detecting inter-turn short-circuit faults in rotor windings using wavelet transformation and empirical mode decomposition. A MATLAB/Simulink model is developed based on electrical parameters to simulate the inter-turn short circuit by adding a resistor parallel to phase “a” of the rotor. The resulting high current in the new phase indicates the presence of the short circuit. By measuring the rotor and stator three-phase currents, the fault can be detected as the currents exhibit asymmetric behavior. Fluctuations in the electromagnetic torque also occur during the fault. The wavelet transform is applied to the rotor current, revealing an effective analysis of sideband frequency components. Specifically, changes in amplitude and frequency, particularly in d7 and a7, indicate the presence of harmonics generated by the inter-turn short circuit. The simulation results demonstrate the effectiveness of wavelet transformation in analyzing these frequency components. Additionally, this study explores the use of empirical mode decomposition to detect faults in their early stages, observing substantial changes in the instantaneous amplitudes of the first three intrinsic mode functions during fault onset. The proposed technique is straightforward and reliable, making it suitable for application in wind turbines with simple electrical inputs.

## 1. Introduction

Induction motors, which account for approximately 50% of the total electricity consumed worldwide [1], are extensively utilized electrical devices. They serve as the foundation of industrial production and play a crucial role in countries’ economic wellbeing. Moreover, induction generators are increasingly employed in environmentally friendly applications like the harnessing of wind [2], hydro [3], and tidal energy [4]. Despite their reputation for affordability, reliability, and sturdiness, induction motors often operate under adverse conditions, including high ambient temperatures, elevated humidity levels, and overloads. These conditions can result in malfunctions, significant maintenance expenses, and unexpected shutdowns, causing financial losses [5,6]. Consequently, both the scientific and industrial communities are actively working towards developing systems that ensure the optimal performance of induction motors, with the aim of minimizing maintenance and inspection costs [7,8,9,10]. Among the various failure types that affect induction motors, electrical and mechanical faults are the most commonly encountered.

Sophisticated techniques for monitoring the health of induction motors and minimizing maintenance costs are necessary due to the fluctuating demands of the electricity network. Based on available data, the most common types of faults affecting induction motors are rotor-related faults (10%), stator-coil-related faults (40%), and other faults, such as end-ring faults (12%) [11,12,13]. When a coil experiences a short circuit, it leads to the generation of a higher current in that particular coil, causing rapid heating. This heat damages the insulation layers of adjacent wires and exacerbates the extent of failure [14]. Early detection is crucial to prevent the escalation of faults and decrease the risk of system failures. Some severe faults, such as phase-to-phase and phase-to-ground short-circuit faults, originate from inter-turn short-circuit (ITSC) faults [15,16]. Nonetheless, the identification of ITSC faults poses significant obstacles [17], and as the operational duration extends, the harm escalates gradually, leading to increasingly severe outcomes.

Existing research in fault detection for induction machines has explored several diagnostic techniques. Studies have focused on detecting faults in both stator and rotor windings of doubly fed induction machines (DFIMs) [18,19,20,21,22,23]. Some researchers have utilized neural-network-based modeling of vibration spectra to address electrical faults in the stator winding [24]. Other works have employed magnetic flux and vibration analysis techniques to identify specific fault frequencies in three-phase asynchronous motors [25]. In the context of wound-rotor induction machines (WRIMs), a novel strategy examined the magnetic field near the motor, utilizing fast Fourier transform and discrete wavelet transform (DWT) to analyze the stator current [26]. Additionally, DWT has been utilized to diagnose imbalances in both the stators and rotors of WRIMs, eliminating the need for slip assessment [27]. For doubly fed induction generators (DFIGs), a combination of DWT and the time-synchronous averaging algorithm was employed to identify inter-turn short-circuit faults [28]. Furthermore, a novel approach using the Hilbert transform was suggested to identify defects in inter-turn short-circuit faults [29]. Additionally, promising diagnostic techniques such as acoustic emission analysis [30,31] and vibration signals [32,33,34] have been utilized for identifying various issues, including broken bars, bearing failures, voltage imbalances, and partial discharges.

This paper presents a novel approach for efficient fault detection of rotor minor inter-turn short-circuit faults in induction machines. The main innovation lies in the integration of wavelet transform and empirical mode decomposition (EMD) for detecting these minor faults, particularly in their early stages. This unique methodology offers enhanced sensitivity and accuracy, enabling the identification of subtle inter-turn short-circuit faults that often go undetected by conventional techniques. The key contributions include the development of a new detection method, early fault detection capability, and practical applicability to wind turbines with simple electrical inputs. Through comprehensive simulations and analyses, the effectiveness and reliability of the proposed technique are demonstrated, emphasizing its potential significance for fault detection in induction machines, specifically in the context of rotor minor inter-turn short-circuit faults. The rest of this article is structured into the following sections: Section 2 introduces the suggested mathematical model of the machine. Subsequently, in Section 3, the fault detection results and analysis methods are discussed. Lastly, Section 4 provides the conclusions.

## 2. Mathematical Modeling of the Machine

The DFIM comprises a stator winding and a rotor winding equipped with slip rings. The stator consists of three-phase insulated windings designed to form the desired pole configuration, and it is connected to the grid through a three-phase transformer. Similarly, the rotor also consists of three-phase insulated windings connected to an external stationary circuit via slip rings and brushes. These components enable controlled rotor current injection or absorption from the rotor windings.

### 2.1. Machine Model in a-b-c Coordinates

The structure of the analyzed induction machine includes three identical phase windings on the stator, arranged at 120 electric degrees of phase difference configuration, and three identical phase windings on the rotor with a similar phase difference. The machine has a constant air gap (close slots in an ideal approach) and an unsaturated (linear) magnetic circuit, allowing each winding to be characterized by a main and leakage inductance. Each phase winding has 228 turns on the stator and 100 turns on the rotor, with a harmonic distribution, and all inductances are considered to be constant.

**Figure 1:**
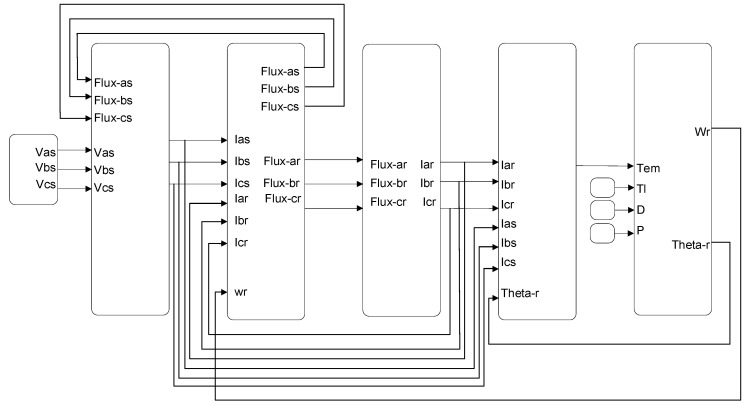
An illustrative diagram of the DFIG model created using MATLAB/Simulink.

The machine model is developed in the natural a-b-c coordinates, using its electrical parameters, including resistances, leakage, self, and mutual inductances of the rotor and stator. The state-space machine model is given in Equations (1) and (2), which originate from Kirchhoff’s voltage law [35]. An illustrative diagram of the DFIG model, created using MATLAB/Simulink, is presented in Figure 1.

(1) $$\frac{d}{dt}\left[\begin{array}{c} \left[i_{S}\right]\\ \left[i_{R}\right] \end{array}\right]={\left[\begin{array}{cc} \left[L_{SS}\right] & \left[L_{SR}\right]\\ \left[L_{RS}\right] & \left[L_{RR}\right] \end{array}\right]}^{-1}\left[\begin{array}{cc} \left[R_{S}\right] & \omega_{r}\frac{d}{d\theta_{r}}\left[L_{SR}\right]\\ \omega_{r}\frac{d}{d\theta_{r}}\left[L_{RS}\right] & \left[R_{R}\right] \end{array}\right]\left[\begin{array}{c} \left[i_{S}\right]\\ \left[i_{R}\right] \end{array}\right]+{\left[\begin{array}{cc} \left[L_{SS}\right] & \left[L_{SR}\right]\\ \left[L_{RS}\right] & \left[L_{RR}\right] \end{array}\right]}^{-1}\left[\begin{array}{c} \left[v_{S}\right]\\ \left[v_{R}\right] \end{array}\right]$$

(2) $$\left[\begin{array}{c} \left[i_{S}\right]\\ \left[i_{R}\right] \end{array}\right]=\left[\begin{array}{cc} \left[I\right] & \left[0\right]\\ \left[0\right] & \left[I\right] \end{array}\right]\left[\begin{array}{c} \left[i_{S}\right]\\ \left[i_{R}\right] \end{array}\right]$$

where *R*, *L*, *v*, and *i* represent the resistance, inductance, voltage, and current, respectively; *θ* and *ω* represent the angle of the rotor and the electrical angular speed, respectively. The subscripts *R*, *S*, *RS*, and *SR* represent a rotor, a stator, a stator-to-rotor quantity, and a rotor-to-stator quantity, respectively.

### 2.2. Model of Inter-Turn Short-Circuit Fault

In this article, we considered faulted turns as two, three, or five turns, which are indicative of “minor faults” due to their limited impact on the overall winding. Additionally, interpreting the faulted turns as percentages, such as 2%, 3%, or 5% of the total number of turns in the phase (given that there are 100 turns in the rotor winding), also categorizes them as “minor faults”, since they constitute a relatively small proportion of the total turns. Detecting and addressing these “minor faults” at an early stage is crucial, as they have the potential to escalate into more severe issues, leading to significant damage and possible system failures.

**Figure 2:**
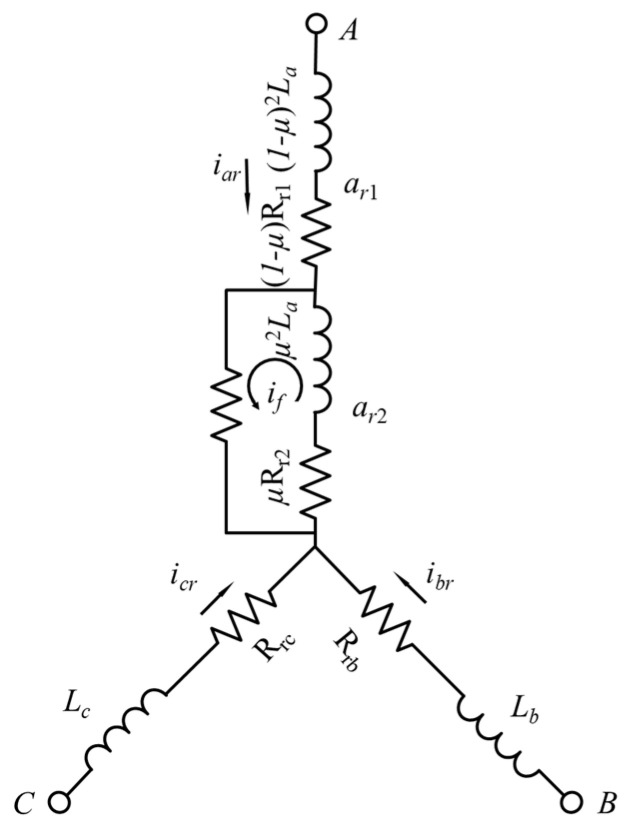
Inter-turn short-circuit fault in the rotor winding.

The winding configuration of a DFIM with a rotor phase “a” short-circuit fault is depicted in Figure 2. The fault leads to the division of the faulty phase into two distinct parts: the shorted turns (ar2) and the un-shorted turns (ar1). Notably, the shorted turns form a closed-loop circuit, effectively representing a new phase of the rotor. To derive the necessary models, several assumptions must be considered, including uniform spatial displacement and an equal number of turns for each rotor phase, sinusoidal distribution of both the stator and rotor windings, operation at an unsaturated point, exclusion of skin or slot effects, and negligible insulation break resistance.

The occurrence of an ITSC fault is attributed to the short-circuiting of two tapping points. When such a fault happens in phase “a”, a new phase “d” is introduced into the circuit of the rotor winding. This addition results in changes to the relevant phase inductance and resistance. The extent of the shorted turn is quantified by the parameter μ, defined as the number of faulted turns per the total number of turns in that phase [36]. The self-inductance of the faulted phase is decreased proportionally to the square of one minus the fault part μ, as given in Equation (3). Additionally, the mutual inductance between the fault-free and faulted phases is defined by Equation (4), and the resistance of the faulted phase is specified in Equation (5).

(3) $$L_{R,faulted}={\left(1-\mu \right)}^{2}L_{R}$$

(4) $$L_{m,faulted}=\left(1-\mu \right)L_{m}$$

(5) $$R_{R,faulted}=\left(1-\mu \right)R_{R}$$

Similarly, for short-circuited turns, the self-inductance increases in square to their number, as defined in Equation (6). The mutual inductance between the fault-free phases and short-circuited turns increases linearly with their number, as shown in Equation (7). Equation (8) is the relation of the resistance in the case of the short-circuited turns. Here, the value of *μ* depends on the number of shorted turns. For example, if two turns of phase “a” are shorted and the total number of turns in phase “a” is 100, then the value of *μ* will be 0.02.

(6) $$L_{fault}=\mu^{2}L_{R}$$

(7) $$L_{m,fault}=\mu L_{m}$$

(8) $$R_{fault}=\mu R_{R}$$

With these assumptions, resistance, self-inductance, and the mutual inductance of the machine can be calculated from Equations (9) to (12).

(9) $$\left[\begin{array}{c} R_{s}\\ R_{r} \end{array}\right]=\left[\begin{array}{ccccccc} r_{s} & 0 & 0 & 0 & 0 & 0 & 0\\ 0 & r_{s} & 0 & 0 & 0 & 0 & 0\\ 0 & 0 & r_{s} & 0 & 0 & 0 & 0\\ 0 & 0 & 0 & \left(1-\mu \right)r_{r} & 0 & 0 & 0\\ 0 & 0 & 0 & 0 & r_{r} & 0 & 0\\ 0 & 0 & 0 & 0 & 0 & r_{r} & 0\\ 0 & 0 & 0 & 0 & 0 & 0 & \mu r_{r} \end{array}\right]$$

(10) $$L_{SS}=\left[\begin{array}{ccc} L_{s} & -\frac{1}{2}L_{ms} & -\frac{1}{2}L_{ms}\\ -\frac{1}{2}L_{ms} & L_{s} & -\frac{1}{2}L_{ms}\\ -\frac{1}{2}L_{ms} & -\frac{1}{2}L_{ms} & L_{s} \end{array}\right]$$

(11) $$L_{RR}=\left[\begin{array}{cccc} {\left(1-\mu \right)}^{2}L_{r} & -\frac{1}{2}\left(1-\mu \right)L_{mr} & -\frac{1}{2}\left(1-\mu \right)L_{mr} & \left(1-\mu \right)\left(\mu \right)L_{mr}\\ -\frac{1}{2}\left(1-\mu \right)L_{mr} & L_{r} & -\frac{1}{2}L_{mr} & -\frac{1}{2}\left(\mu \right)L_{mr}\\ -\frac{1}{2}\left(1-\mu \right)L_{mr} & -\frac{1}{2}L_{mr} & L_{r} & -\frac{1}{2}\left(\mu \right)L_{mr}\\ \left(1-\mu \right)\left(\mu \right)L_{mr} & -\frac{1}{2}\left(\mu \right)L_{mr} & -\frac{1}{2}\left(\mu \right)L_{mr} & {\left(\mu \right)}^{2}L_{r} \end{array}\right]$$

(12) $$L_{SR}=L_{SR}{}^{T}=L_{sr}\left[\begin{array}{cccc} \left(1-\mu \right)\cos\theta_{r} & \cos\left(\theta_{r}+\frac{2}{3}\pi \right) & \cos\left(\theta_{r}-\frac{2}{3}\pi \right) & \left(\mu \right)\cos\theta \\ \left(1-\mu \right)\cos\left(\theta_{r}-\frac{2}{3}\pi \right) & \cos\theta_{r} & \cos\left(\theta_{r}+\frac{2}{3}\pi \right) & \left(\mu \right)\cos\left(\theta_{r}-\frac{2}{3}\pi \right)\\ \left(1-\mu \right)\cos\left(\theta_{r}+\frac{2}{3}\pi \right) & \cos\left(\theta_{r}-\frac{2}{3}\pi \right) & \cos\theta_{r} & \left(\mu \right)\cos\left(\theta_{r}+\frac{2}{3}\pi \right) \end{array}\right]$$

where *L*_s_ = *L_ms_* + *L_ls_* and *L*_r_ = *L_mr_* + *L_lr_*.

Below is the expression for the electromagnetic torque produced by the machine:

(13) $$T_{e}=\frac{1}{2}n_{p}\left[i_{abcdr}^{T}\frac{\partial L_{rs}}{\partial \theta_{r}}i_{abcs}+i_{abcs}^{T}\frac{\partial L_{sr}}{\partial \theta_{r}}i_{abcdr}\right]$$

The classical differential equation provided in Equation (14) is employed to simulate the mechanical component.

(14) $$T_{e}-T_{m}=\frac{J}{n_{p}}\frac{d\omega_{r}}{dt}+\frac{D\omega_{r}}{n_{p}}$$

where *T_e_*, *T_m_*, *n*_p_, *ω_r_*, *J*, and *D* are the electromagnetic torque, mechanical torque, number of pole pairs, electrical angular velocity, inertia, and damping coefficient of the machine, respectively.

## 3. Simulation Results and Analysis

**Figure 3:**
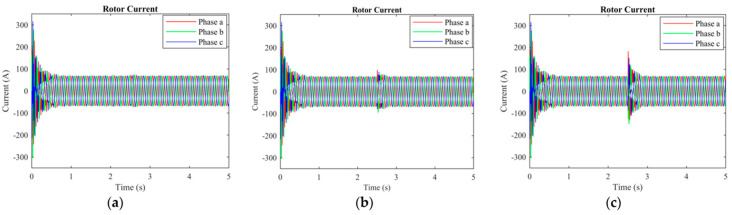
Rotor current of the machine: (**a**) 2 turns are shorted; (**b**) 3 turns are shorted; (**c**) 5 turns are shorted.

**Figure 4:**
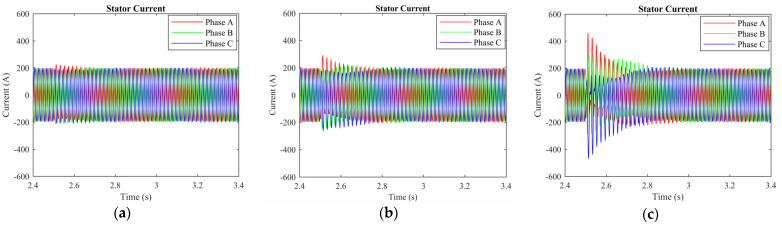
Three phase stator current of the machine: (**a**) 2 turns are shorted; (**b**) 3 turns are shorted; (**c**) 5 turns are shorted.

The rotor current, stator current, electromagnetic torque, wavelet transformation, and EMD analysis were used to differentiate the normal and short-circuit fault conditions. The DFIM model was simulated in MATLAB/Simulink. The rotor speed of the machine was set to 1712 r.p.m, and the time of the machine running was set to 5 s. After 2.5 s, a fault occurred, and it remained for 0.3 s. The machine parameters are stated in Table 1. Three-phase rotor currents for two-, three-, and five-turn short circuits are presented in Figure 3. Three-phase stator currents for two-, three-, and five-turn short circuits are shown in Figure 4. Time-domain analysis showed asymmetrical behavior during the fault. Electromagnetic torque displayed a fluctuation in faulty conditions. Approximation and details of level 7 of the wavelet transformation were found to be effective for analyzing the sideband frequency component. The first three intrinsic mode functions (IMFs) of EMD were found to be useful to detect faults in their early stages.

### 3.1. Harmonic Analysis of Rotor and Stator Currents and Electromagnetic Torque

The presence of harmonics indicates a fault in the machine. When the machine is operating normally, a balanced flow of currents exists between the stator and rotor, resulting in the formation of a magnetic field through the magnetic motive force (MMF).

Figure 3 is the time-domain representation of the rotor current with two, three, and five turns short-circuited in the rotor winding. The three phases of the rotor currents are symmetrical, and their RMS values are equal for the healthy condition of the machine. However, the asymmetrical behavior in the amplitude of the three-phase currents can be seen in Figure 3a–c, and their RMS values are also not equal after 2.5 s. This asymmetrical behavior in the amplitude is due to the harmonics induced when the ITSC fault occurs in the rotor windings. This asymmetrical behavior in a three-phase rotor current is more obvious when the number of shorted turns is higher.

When the doubly fed wind generator is operating correctly, the magnetic field formed by the rotor current and stator current remains relatively stable, creating a closed loop that passes through the rotor, air gap, and stator core. The magnetic field of the motor is evenly distributed around the virtual north and south poles, resulting in a balanced configuration. During this normal state of operation, the external magnetic field of the motor’s stator core is almost nonexistent. However, if a short circuit occurs in the rotor winding, the symmetry of the motor’s three-phase windings is disrupted. This leads to the addition of high-order harmonic components in the magnetic field of the air gap, which can impact the stator current. Observations show that a short circuit in the rotor winding affects the stator windings, as evidenced by changes in the stator current after the occurrence of such a short circuit. Figure 4a–c illustrate this phenomenon for scenarios where two, three, and five turns are shorted in the rotor winding. Under normal circumstances, the three-phase stator currents are symmetrical, with their root mean square (RMS) values being equal.

**Figure 5:**
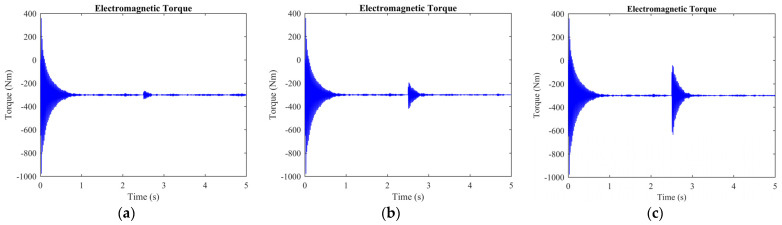
Electromagnetic torque of the machine: (**a**) 2 turns are shorted; (**b**) 3 turns are shorted; (**c**) 5 turns are shorted.

Figure 5 depicts the variations observed in the electromagnetic torque across various scenarios. The torque remains stable during regular machine operation, without any fluctuations. Conversely, the occurrence of a short-circuit fault in the rotor winding, as depicted in Figure 5a–c, generates harmonic components within the electromagnetic torque. Harmonics, which emerge as a result of the fault, manifest as fluctuations or ripples within the torque waveform, introducing additional frequencies.

The aforementioned Figure 5a–c specifically emphasize the heightened presence of these harmonics. The figure depicts short-circuit faults with two, three, and five turns, respectively. This observation implies a direct correlation between the fault severity and the magnitude of the harmonics generated in the electromagnetic torque. The analysis of these harmonics is important in understanding the behavior of the machine under faulty conditions, and it can be used to diagnose and prevent faults from occurring in the future.

### 3.2. Wavelet Transformation Technique

A wavelet refers to a waveform that exhibits oscillations both above and below the x-axis within a specific timeframe, and its average value remains zero. Its significance lies in its ability to analyze non-periodic signals that do not exhibit consistent changes over time. Wavelets are mathematical functions that fulfill the requirements of both time and frequency localization [37].

It is possible to express the DWT using the Ψ*_l_*_,*m*_ terms as follows:

(15) $$D_{l,m}=\sum_{k=1}^{l+1}x\left(k\right)\Psi_{l,m}\left(k\right)$$

The DWT coefficients can be used to represent the signal *x*(*t*) as follows:

(16) $$x(t)=\sum_{m=-\infty }^{\infty }\sum_{l=-\infty }^{\infty }D_{l,m}\Psi_{l,m}$$

where *k* is a discrete variable, while *n* and *m* are scaling variables. The wavelet function is represented by Ψ, which refers to its scaled and shifted versions.

Unlike the short-time Fourier transform (STFT), which utilizes a fixed analysis window length, the wavelet transform employs varying window lengths, using shorter windows for high frequencies and longer windows for low frequencies. This fundamental distinction allows the wavelet transform to possess a multiresolution capability, enabling the variation of frequency resolution according to specific requirements. The ability to adjust the frequency resolution is not present in the STFT, since it relies on a single analysis window with a constant frequency resolution determined by the window width. The multiresolution feature of the wavelet transform can be understood through the theories presented in [38].

The initial step in this process is to break down the original signal into different “scales” by utilizing a wavelet prototype function known as the “mother wavelet”. This procedure includes the examination of frequency by employing a low-frequency variant of the mother wavelet and the analysis of time using a high-frequency variant of the identical wavelet. Let *x*[*n*] symbolize the discrete time signal, where “*n*” denotes the samples. The signal is subjected to decomposition, yielding *c*1[*n*] and *d*1[*n*] at the first scale. In this decomposition, *ca*1[*n*] signifies the smoothed rendition of the original signal, whereas *cd*1[*n*] corresponds to the detailed rendition.

(17) $$ca1[n]=\sum_{k=1}^{l+1}h\left(k-2n\right)x\left(k\right)$$

(18) $$cd1[n]=\sum_{k=1}^{n+1}g\left(k-2n\right)x\left(k\right)$$

**Figure 6:**
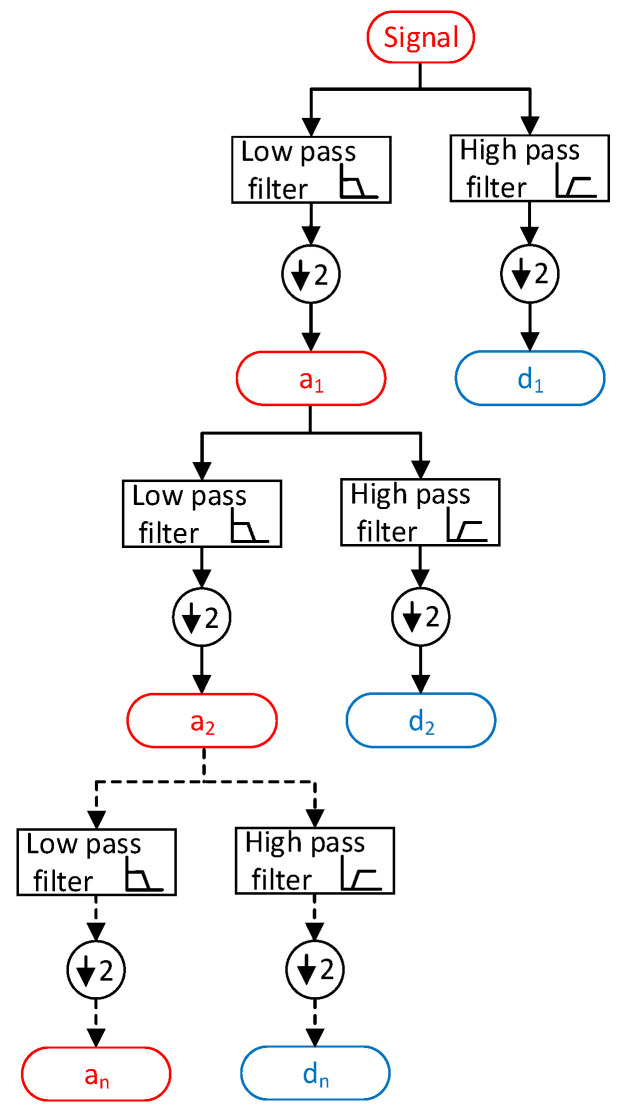
Discrete wavelet decomposition algorithm.

The filter coefficients *h*[*n*] and *g*[*n*] are used to decompose a signal, *x*[*n*], into its approximations, *ca*1[*n*], and details, *cd*1[*n*], at the first scale. The process of decomposition can be repeated at the next higher scale, where the approximation *ca*1[*n*] is decomposed into its approximations, *ca*2[*n*], and details, *cd*2[*n*], and so on. This iterative process results in a multiresolution representation of the original signal, with each level providing a lower resolution and more compact representation. The detail coefficients, *cd*1[*n*], can be used to recognize instances of major changes or noise in the signal by applying a thresholding technique [39]. The decomposition algorithm of discrete wavelet transformation is explained in Figure 6.

The choice of Daubechies-44 as the mother wavelet in the analysis was based on its high energy content. To effectively monitor the low-frequency elements, the appropriate number of decomposition levels was determined based on [40]. This resulted in a calculated value of six levels, taking into account a sampling rate of 5000 samples per second and a stator frequency of 50 Hz. Typically, the level of decomposition for the detail signal needs to be equal to or greater than that of the frequency components generated by rotor eccentricities or irregularities. The frequency bands corresponding to the wavelet signal can be found in Table 2.

**Figure 7:**
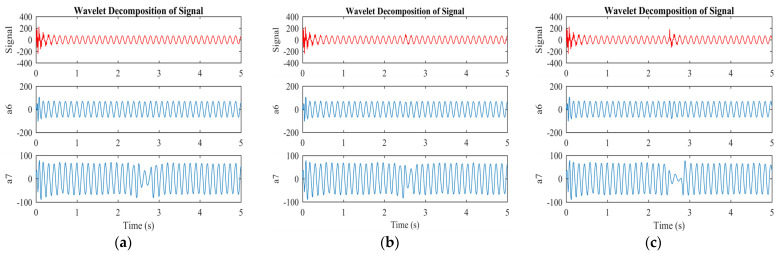
Approximate signal of wavelet transformation under different conditions of the machine: (**a**) 2 turns are shorted; (**b**) 3 turns are shorted; (**c**) 5 turns are shorted.

**Figure 8:**
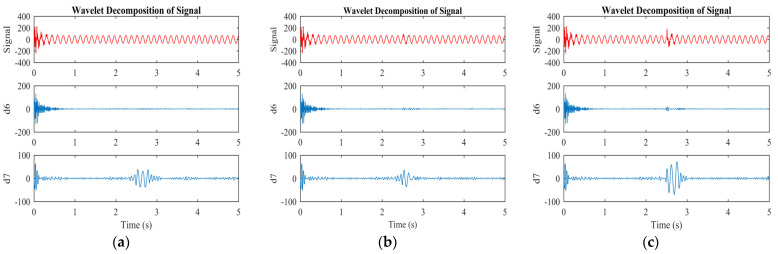
Detailed signals of wavelet transformation under different conditions of the machine: (**a**) 2 turns are shorted; (**b**) 3 turns are shorted; (**c**) 5 turns are shorted.

Figure 7 and Figure 8 illustrate the original, approximate, and detailed signals for levels six and seven of wavelet analysis of the machine under faulty conditions. The approximate information is derived from the low-pass filter, while the detailed signal information is derived from the high-pass filter. The approximate portion of the signal indicates the time of the fault. The approximate and detailed signals for level 7 are repeatedly applied until the required level of the signal is achieved, whereas the detailed signal remains unchanged after the initial division. These approximate and detailed signals indicate the appropriate levels for identifying the harmonic components in the rotor current. The amplitude and frequency of the wavelet signals remain unchanged (a6, a7, d6, and d7) during normal machine operation.

However, in the event of an ITSC fault, the frequency of the approximate signal (a7) changes, and the detailed signal (d7) experiences an increase in both frequency and amplitude, as shown in Figure 6a and Figure 7a for two shorted turns. These variations in frequency and amplitude in the approximate (a7) and detailed (d7) signals are due to the harmonics induced in the rotor currents as a result of the ITSC fault in the rotor winding. The approximate (a6) and detailed (d6) signals do not exhibit changes in their frequency or amplitude, indicating that level seven is the most appropriate level for detecting faults. The same trend can be observed for three and five shorted turns.

### 3.3. Empirical Mode Decomposition Analysis

EMD is a novel analysis method in the time–frequency domain. It was developed by Huang and colleagues [41] and possesses the advantageous characteristic of robustness when dealing with nonlinear and nonstationary data. EMD adaptively decomposes a signal into a sequence of frequency components, known as IMFs, based on the signal’s characteristics. By utilizing the Hilbert spectrum, the instantaneous frequency and instantaneous amplitude of each IMF layer’s empirical mode component can be obtained, allowing for the analysis of the current signal and the observation of changes in IMF amplitude. In this paper, the EMD technique is proposed for application to the current signal to detect the rotor ITSCF.

The current signal of the motor comprises multiple oscillation modes, which EMD separates according to the different frequency segments of these modes. Each IMF represents a distinct frequency range and characterizes the oscillation mode within the current signal. Only signals that meet specific conditions can be considered to be IMF components of the decomposed signal. These conditions include the absolute value of the difference between the number of extreme points in the signal and the number of zero crossings that are less than or equal to one, with the small-value envelope being symmetrical about the x-axis. Any signal decomposed using EMD can be expressed as the sum of a series of IMF components and a residual term, as shown in Equation (19), where *c_j_*(*t*) represents the jth mode function and r is the residual.

(19) $$i(t)=\sum_{j=1}^{n}c_{j}(t)+r_{n}(t)$$

**Figure 9:**
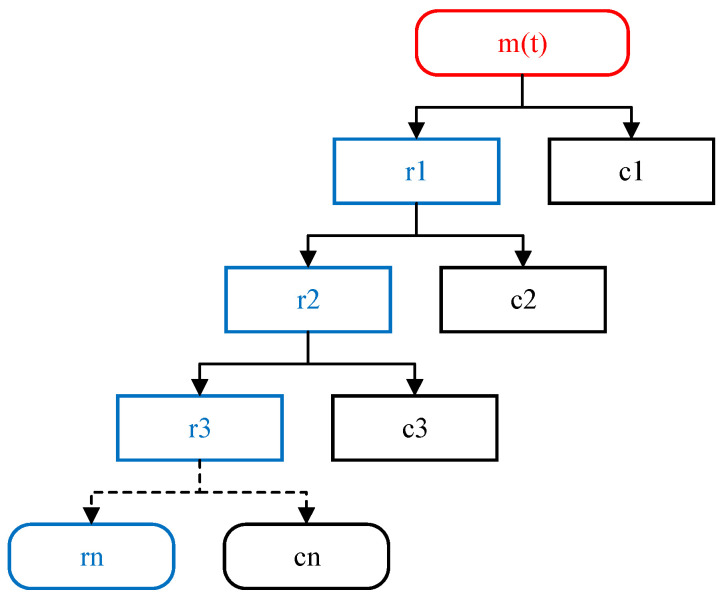
Empirical mode decomposition filtering features.

The main steps of EMD are as follows:

1. The original signal can be accurately approximated by employing a cubic spline function to fit its upper and lower envelopes by calculating the average value of these envelopes, denoted as *m*_1_(*t*);
2. Calculating the difference between the original signal and *m*_1_(*t*), and recording it as *h*_1_(*t*);
3. Repeating the above two steps until the obtained signal *h*_1_(*t*) satisfies the requirements of an IMF component. At this point, the obtained signal is the first-layer empirical mode component, denoted as *c*_1_(*t*), which primarily contains high-frequency harmonics from the original signal;
4. Finding the variance between the sum of the original signal c_1_(*t*) and recording it as a new original signal. Repeating the above three steps for this signal to obtain all IMF components. The remaining signal is a monotone function with no extreme values, which is recorded as the residual term *r_n_*(*t*). In this study, the original signal was iteratively decomposed six times, resulting in six IMFs. The filtering characteristics of EMD are illustrated in Figure 9.

Empirical mode decomposition (EMD) provides information about the internal structure of a signal. IMF1 represents the highest-frequency components of the original signal, followed by IMF2, IMF3, and so on. On the other hand, the residue “r” represents the overall trend of low-frequency components.

When applying EMD to the current in the rotor winding of the ITSCF system, six layers of empirical modal components (IMFs) are obtained. IMF1, IMF2, and IMF3 exhibit abrupt changes in amplitude at the onset of a fault. This characteristic can be utilized to identify short-circuit faults in the stator winding of the generator. The time-domain analysis reveals significant variations in the instantaneous amplitudes of the IMF1, IMF2, and IMF3 components.

**Figure 10:**
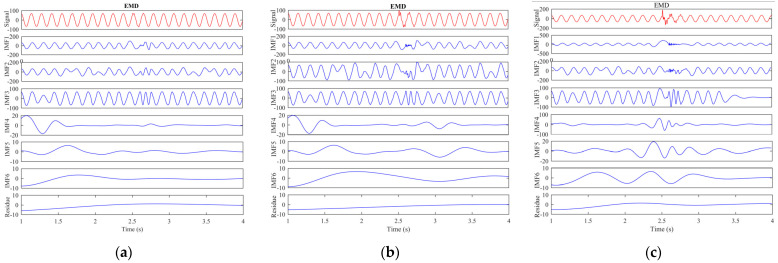
Empirical mode decomposition analysis: (**a**) 2 turns are shorted; (**b**) 3 turns are shorted; (**c**) 5 turns are shorted.

Based on the data presented in Figure 10, the presence of a short circuit in the generator’s rotor winding leads to a breakdown of the fault-phase current through EMD. This breakdown results in a sudden change in the amplitudes of IMF1, IMF2, and IMF3. This occurrence is due to EMD separating the original signal into different frequency components. When a short-circuit fault occurs in the rotor winding, a new inter-turn current arises within the short-circuit loop. Consequently, the signal envelope in these frequency ranges changes, incorporating fault-related details. The significant alterations observed in the IMF1, IMF2, and IMF3 components during the fault can be attributed to the generator fault. Therefore, even though a minor short-circuit fault might not cause a substantial variation in the generator’s rotor current, EMD proves to be an effective technique for fault detection in the generator. Figure 10 demonstrates that the peak-to-peak values of IMF1, IMF2, and IMF3 increase as the fault level escalates.

## 4. Conclusions and Future Work

Inter-turn short-circuit (ITSC) situations play a vital role in the operation of doubly fed induction generators (DFIGs). In this study, the proposed approach using wavelet transformation and empirical mode decomposition (EMD) was effective in detecting ITSC faults in rotor windings. A mathematical model of the DFIG was developed, incorporating a resistor with a resistance of 0.07 ohms in parallel to phase “a” of the rotor winding to simulate the ITSC. The resulting high current in the new phase served as an indicator of the fault. The analysis of rotor and stator three-phase currents proved useful for detecting the presence of ITSC faults, as the currents exhibited asymmetric behavior during the fault. An analysis of the results obtained by the two methods, wavelet transformation and empirical mode decomposition (EMD), revealed that wavelet transformation is more efficient in fault detection. The signature of wavelet signals, particularly in components a6, a7, d6, and d7, proved to be more prominent and distinct during the occurrence of inter-turn short-circuit (ITSC) faults in the rotor winding. On the other hand, while EMD also provided valuable insights into the signal structure, the changes in the IMF1, IMF2, and IMF3 components were not as pronounced as those observed in the wavelet analysis. Therefore, based on the results, wavelet transformation can be considered to be more effective in identifying and diagnosing ITSC faults in the generator, due to its clear and prominent fault signatures. Future work should be dedicated to exploring additional diagnostic techniques, such as multivariate statistical analysis, which is widely applied for detecting rotor minor inter-turn short circuits (i.e., incipient faults) in induction machines.

## Figures and Tables

**Table 1:** Parameters of the machine.

Variable	Rating Values	Variable	Rating Values
Stator resistance	0.024 Ω	Rotor resistance	0.037 Ω
Stator inductance	0.074 Ω	Rotor inductance	0.087 Ω
Magnetizing inductance	4.06 Ω	Stator voltage	380 v
Inertia	2.45 kg·m^2^	Rated load torque	530 N·m
Rotor open-circuit voltage	1092 V	Stator frequency	50 Hz
Stator turns	228	Rotor turns	100

**Table 2:** The frequency bands of each level.

Approximations (a)	Frequency Bands (Hz)	Details (d)	Frequency Bands (Hz)
a_1_	(0–2500)	d_1_	(2500–5000)
a_2_	(0–1250)	d_2_	(1250–2500)
a_3_	(0–625)	d_3_	(625–1250)
a_4_	(0–312.5)	d_4_	(312.5–625)
a_5_	(0–156.25)	d_5_	(156.25–312.5)
a_6_	(0–78.125)	d_6_	(78.125–156.25)
a_7_	(0–39.062)	d_7_	(39.062–78.125)

## Data Availability

Not applicable.
